# Supplementary figures and images for: BHRF1 Enhances EBV Mediated Nasopharyngeal Carcinoma Tumorigenesis through Modulating Mitophagy Associated with Mitochondrial Membrane Permeabilization Transition
Source: Cells. 2020 May 7;9(5):1158. doi: 10.3390/cells9051158 (PMC7290790; doi:10.3390/cells9051158)

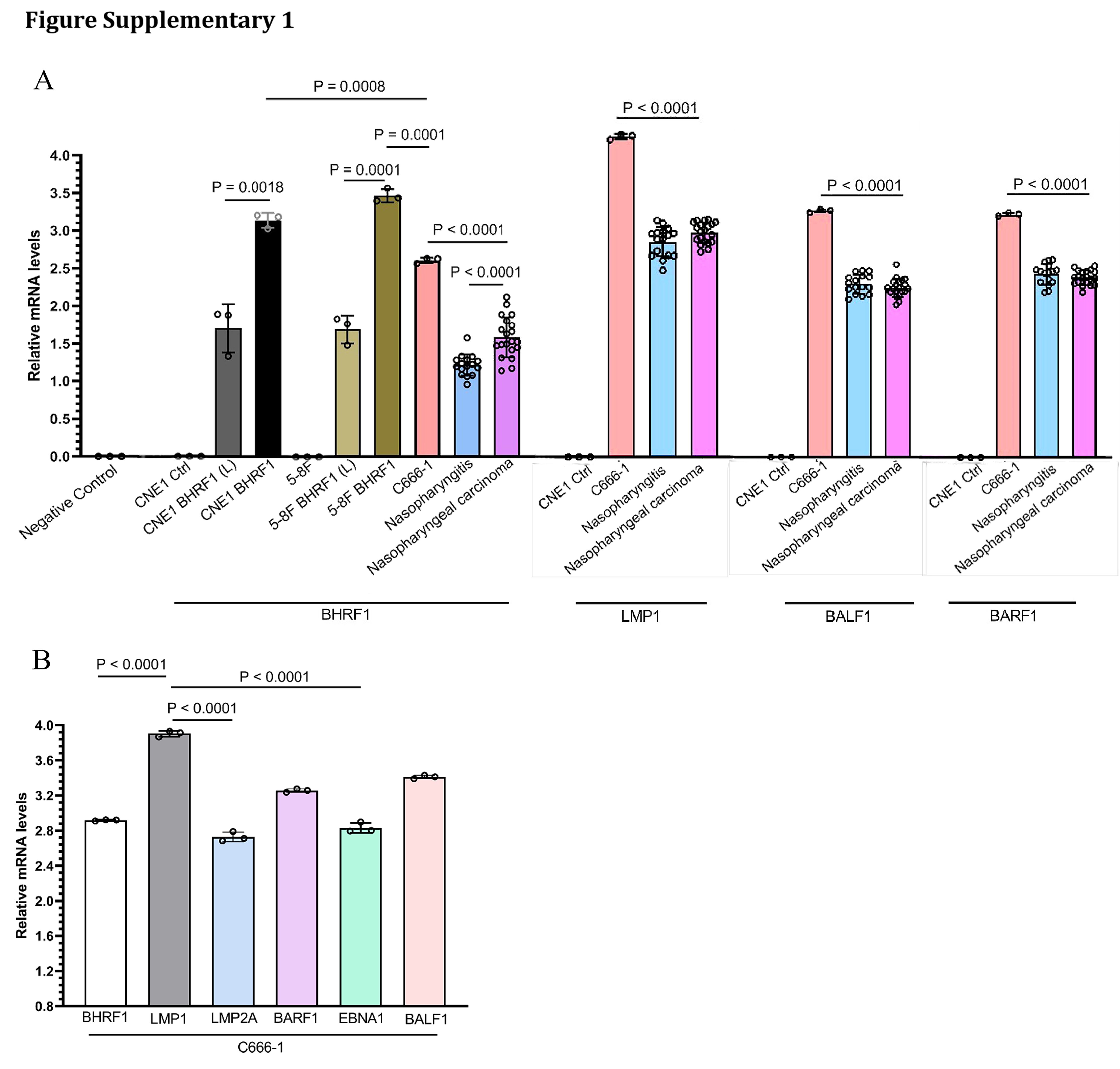

Supplement: Supplementary file 1 [file cells-09-01158-s001.zip › Supplementart Figure S1.png]

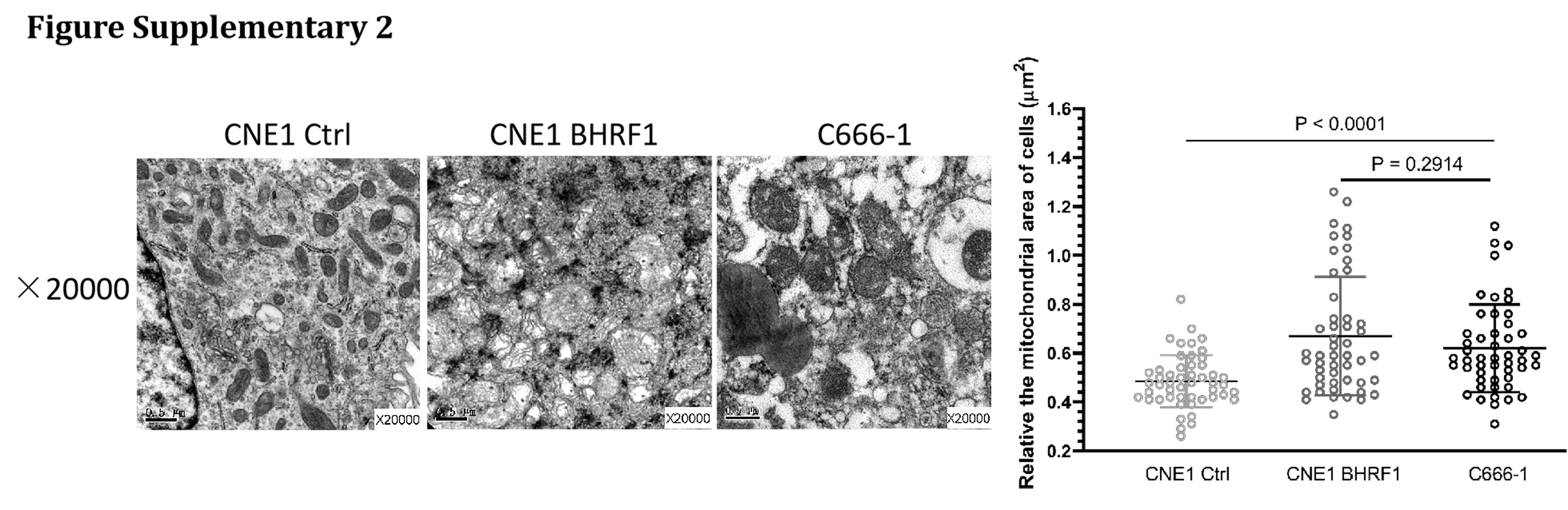

Supplement: Supplementary file 1 [file cells-09-01158-s001.zip › Supplementart Figure S2.png]
